# Structuring supplemental materials in support of reproducibility

**DOI:** 10.1186/s13059-017-1205-3

**Published:** 2017-04-05

**Authors:** Dov Greenbaum, Joel Rozowsky, Victoria Stodden, Mark Gerstein

**Affiliations:** 1 Zvi Meitar Institute for Legal Implications of Emerging Technologies, Radzyner Law School, Interdisciplinary Center, Herzliya, Israel; 2 Program in Computational Biology and Bioinformatics, Yale University, New Haven, CT 06520 USA; 3 Integrated Graduate Program in Physical and Engineering Biology, Yale University, New Haven, CT 06520 USA; 4 Department of Molecular Biophysics and Biochemistry, Yale University, New Haven, CT 06520 USA; 5 Department of Computer Science, Yale University, New Haven, CT 06520 USA; 6 Graduate School of Library and Information Science, University of Illinois at Urbana-Champaign, 501 E Daniel St, Champaign, IL 61820 USA

## Abstract

Supplements are increasingly important to the scientific record, particularly in genomics. However, they are often underutilized. Optimally, supplements should make results findable, accessible, interoperable, and reusable (i.e., “FAIR”). Moreover, properly off-loading to them the data and detail in a paper could make the main text more readable. We propose a hierarchical organization for supplements, with some parts paralleling and “shadowing” the main text and other elements branching off from it, and we suggest a specific formatting to make this structure explicit. Furthermore, sections of the supplement could be presented in multiple scientific “dialects”, including machine-readable and lay-friendly formats.

**Electronic supplementary material:**

The online version of this article (doi:10.1186/s13059-017-1205-3) contains supplementary material, which is available to authorized users.

## Introduction

Journal article supplements (also known as “additional files” or supplementary materials) are an increasingly indispensable resource for researchers. They should be designed to provide essential metadata and documentation and act as stand-alone repositories for small data sets. Unfortunately, they often fail to live up to these responsibilities. In his “Stories from the Supplement” lecture [1], Lior Pachter elegantly described many of these missed opportunities, including where ideas are often contained entirely within the supplement and are difficult to find from the main text. (Please see Additional file 1 for further details; as described herein, this mirrors and expands upon the hierarchy of this paper.)

Supplements contain a tremendous amount of information, including facts and analyses associated—sometimes only tenuously—with the corresponding published papers. Occasionally, entire projects are inaccessibly buried within [1]. With some articles having supplements ballooning to multiple times the length of the paper itself [2, 3], the data within becomes nearly impossible to find. The editing of supplements, which is often poor, exacerbates these issues. Further damage is caused when researchers, fearful of burying relevant data in inaccessible supplements, increasingly cram more data into their papers, eschewing the vernacular in favor of terse, incoherent terminology. As a result, some scientific papers have become more convoluted and unintelligible.

With all these problems, many are calling to curb the use of supplements [4, 5]. We believe this to be shortsighted. Instead, enforcing a considered and standardized approach would make supplements an effective and indispensable tool.

## Proposal

Supplements have the potential to provide substantial clarity to the published text, not only by providing much-needed annotation, but also additional information and data. Even though the supplement will likely never be as precise or as defined as the main text, considerable improvements need to be made across the board. Without the constraints of space, online supplemental material can afford to be clearly written, better organized, and well-documented, allowing for an expanded and useful representation of the published research and its results.

**Fig. 1:**
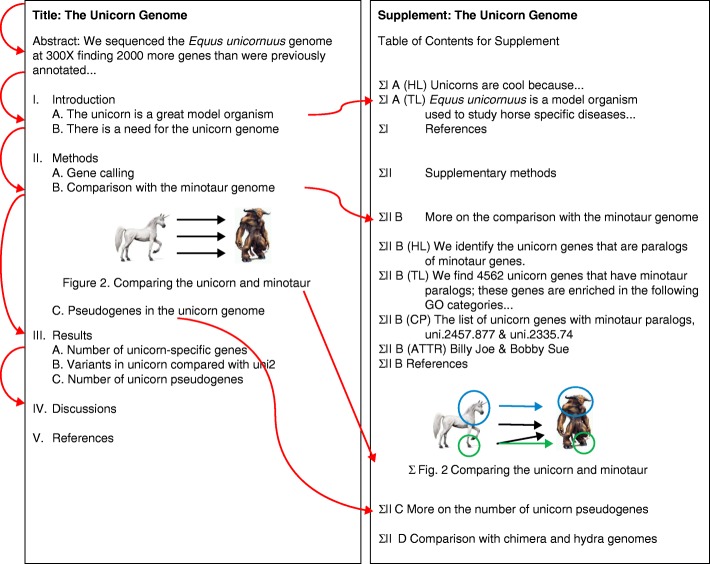
In this figure we present an illustrative example of how the information contained in a structured supplement parallels the layout of the main text of a paper. Each section in the supplement has the prefix ∑, denoting a supplementary section. Supplementary subsections that parallel main subsections are denoted by ∥, while those that are only in the supplement are labeled as ∦. Parallel sections in the supplement can also have multiple alternative versions, such as the “high level” version and the “technical language” version

Universally accepted structures and standards will substantially expand the usefulness of supplemental materials. With an indexed, searchable, and useful supplement, authors need not try to fit so much into the main text of the paper, and this will result in a more coherent and readable main text. Notably, both the published paper and its supplement can benefit from tying each section in the main text to its corresponding expanded supplement section, which contains corresponding raw data and related information through an established, logical, and linked hierarchy within a parallel structure (Fig. 1).

### Proposed hierarchy

Within the proposed hierarchy, the paper, the supplement, and all associated data are each seen as interrelated elements within the larger expansive architecture of a stack or research platform. Thus, the primary text would figuratively sit atop the supplement, synthesizing the supplemental information in broad strokes. Other elements would sit beneath the supplement within the stack, including software, databases, and other elements associated with the research. Local links would point to more detailed descriptions of methods and data located further within the supplemental materials.

The detailed description within the supplement that expands upon top-level primary text should be logically subdivided with each corresponding original paper division addressing a coherent aspect of the analyses. The order of these divisions would map onto the order of appearance within the top-level primary text, allowing researchers to easily move between even a physical printed version of the supplement and the original paper.

In a secondary hierarchical structure, each of these individual divisions may relate to its own—potentially vast—supplementary calculations and data sets. These calculations and data sets would be further linked such that they relate back to each division within the supplement, and then to the top-level primary text. To promote machine readability of the data sets, data associated with the paper should be provided in a standard tabular format (e.g., comma-separated values), and charts, graphs, and other pictorial representations of the data should be decomposable, i.e., accompanied by machine-readable files comprising the underlying data. One can also envision shadow tables and figures, which would parallel those in the main text but provide a more expanded layout, with additional detail (Additional files 1 and 2).

Practically speaking, all data falling within the hierarchy should be localized to a single digital location. When absolutely necessary—for example, with regard to sensitive data—hyperlinks can be provided to outside sources. In some cases, the sheer size of intermediate or non-essential data sets may require that some data reside in an off-site website. Here, authors should guarantee link viability, as has been attempted in other disciplines [6].

### Hierarchical information structures

Reading a scientific text can be analogous to an information retrieval task, wherein a reader first peruses an introductory section and then jumps into a more detailed version of that section. The current structure of a standard scientific manuscript implements a simplified version of this idea: a short yet informative title, a more detailed abstract, a somewhat expanding introduction, a detailed results section with detailed tables, and then a conclusion that applies the details more broadly. The proposed supplement guidelines would expand on this age-old structure, building on this pre-existing hierarchy and providing even more levels of information. In a parallel to the main text, the supplement should shadow the paper, providing more detailed explanations for each part of the main text. This would allow a reader looking for more detail to easily find it and then consult the analogous part of the supplement, which would be similarly situated within the hierarchical structure.

In this methodology, scientific writing would be presented both as a simple hierarchy and, concurrently, as parallel passes at increasingly greater levels of detail. Further, this hierarchy provides an essential roadmap that ought to be familiar across all fields (with well-known section headings such as “Introduction”, “Results”, and other standard research paper headings). It would include standardized headings for easy human and machine readability, with the structured headings directly corresponding to headings in the primary paper. Additionally, the supplementary material should be designed to include ample indexable metadata relating various elements within the hierarchy of the paper.

Employing an apt literary metaphor, the published paper would be akin to a primary source, and the supplement would mirror the annotation (designed to add integral, associated, and tangentially relevant context) and other editorial content on that original text. However, the versatility of the supplement allows it to also be an expansive and sometimes meandering—albeit hierarchically organized—Talmud to the Torah of the succinctly and sometimes cryptically presented published paper.

In some instances, the hierarchical paradigms of a supplement can extend beyond that of a single paper to a whole collection of related papers. This becomes all the more relevant as a result of “big consortia science”, in which research projects result in high level papers and a succession of more detailed, related papers, often across multiple journals. Here, all papers can conform to a single global hierarchy with a top-level main paper and more detailed companions [7]. This, in turn, corresponds to various interconnected supplements associated with each individual paper, similar, for example, to the structure of the ENCODE rollout [8]. Importantly, this would help illuminate the interconnectivity of individual papers within a series.

### The FAIR standards: findable, accessible, interoperable and reusable

Employing the FAIR approach for scientific information is essential for guiding the construction of supplements [9]. Data should be: (i) findable, both for human researchers and computers, requiring unique and persistent identifiers (e.g., those provided by groups such as Consortia Advancing Standards in Research Administration Information (CASRAI) [10]); (ii) accessible for the long term by using appropriate open licensing for data, code, and workflow information [11, 12]; (iii) interoperable via shared vocabularies, qualified references, and shared vernacular; and (iv) reusable such that both humans and machines can easily use the data for follow-up research or additional computational analysis.

### Provenance

The veracity of research data requires a complete description of the origins of the data, as well as the process by which that data arrived in its current form (for example, any data manipulation such as normalizations) [13]. Provenance allows data quality to be assessed and provides an audit trail that could uncover sources of error, the location of all the data relevant to replicate the results, and the attributions necessary for assessing ownership, copyright, license limitations, any privacy restrictions, and liabilities, if any, ascribed to erroneous data.

### Workflows

Understanding the provenance of a data set can be substantially helped by the inclusion of workflows within the supplement. Supplements should outline, preferably both superficially and in some depth, the individual and collective workflows that produced and employed resources, and the final conclusions [14]. Notably, workflows should be designed to work on at least two levels: as abstract, general methods and as a more specific, schematic representation of a particular computer code. This is an important limitation: workflows should not necessarily include the code itself, as this paradigm regards supplements as an important platform but not a repository of data.

Workflows are especially relevant for in silico analyses, as reproducibility can turn on the ability to recreate the exact parameters employed. Abstract workflows, flowcharts and/or comments on the code and execution infrastructure of the research are necessary [15]. They should employ standardized identifiers that can be used to reference parts of the workflow itself, the relevant data sets and software, or any other information useful for cross-referencing workflows and their components. Alternatively, third party, open-source solutions such as Galaxy [16] could be used, with the supplement providing links to these solutions [17].

### Language in the supplement

The supplement should be readable by both humans and machines, optimally through the use of distinct formalized languages optimized for each audience. Even in the predominantly English-speaking scientific press, research is conveyed in multiple types of language, including simple vernacular language providing a simplistic, top-level understanding; precise, technical terminology necessary to convey methods to experts and to aid in reproducibility; and increasingly, semi-structured English to aid in computer parsing and automatic text retrieval, indexing, summarization, and searches. This language is similar to what has been described for the structured abstract [18, 19] and the structured digital table [20].

Length limitations often preclude the adequate provision of these novel aspects of papers, and they are rarely provided within the main text of a document. Since space is less constrained within the supplement, it is possible to express the same ideas in multiple iterations and forms. In particular, the same idea can be expressed in multiple “language channels” and additional aspects can be introduced. For example, supplements can include relatively simplistic schematic graphics and easy-to-understand, intuitive text, which might be unnecessary for the primary audience of the paper but are necessary to make the information accessible to an increasing number of multidisciplinary outsiders, or even the lay public. Likewise, the supplement could contain paragraphs of excessively precise scientific detail necessary for reproducibility and easier parsing.

To facilitate the use of machine parse-able sections, the supplement would contain a structured glossary connecting all the entities in the paper and their languages; this glossary—which is distinct from a glossary that defines the specific usages of the terms of art used in the paper—would correlate with standard database identifiers. Within the hierarchical structure proposed, many of the headings of the supplement might also employ a highly standardized format, further enabling computer parsing and human usability.

### Citation standards

All references in the supplement should be indexed in standard indexing databases. In some cases, citation systems will need to be broadened to allow pinpointed referencing between the primary and supplemental text. This would allow readers of the primary text to be directed from the main text to the relevant section in the supplement, and vice versa, using micro-digital object identifiers (DOIs) or other referencing systems. To some degree, this can be accomplished through the hierarchical structure and further simplified through a standardized numbering system, allowing for DOIs of sections, subsections, and even further divisions if necessary. This citation standard can include additional information relating to super-sections, tying together published papers across multiple journals.

With an established hierarchy, different components of the paper and its supplement can be intelligently referenced: clever use of prefixes and suffixes can provide DOI (or similar systems) links to important portions within the supplement.

Unlike the published text, authors can further take advantage of the nature of the supplementary section to micro-reference micro-authorship, utilizing open researcher and contributor IDs (ORCIDs) or other persistent unique identifiers to note which specific author contributed to each portion of the paper. Not only would this provide more realistic accreditation to authors than standard author listings, but it would give interested readers direct access to the appropriate author for the particular area, text, or figure of interest, perhaps through published email addresses.

Figures would not only include captions and links to relevant parts of the text, but might also include additional information related to the relevant contact individuals for each figure and access to the source code and data that generated the figure. Again, this would be particularly important given the growing trend to list tens if not hundreds of authors on genomics papers.

Supplementary material should also include an expanded bibliography, which can be designed to provide contextual information, both with regard to the paper itself and the supplementary material. Furthermore, the bibliography can be annotated to provide substantive information as to how each source relates to the presented information. It may be useful to have separate bibliographies for each section of the supplement, although notably, such citations will likely not yet count as official citations.

## Conclusions

The age of “big data” and “supersized papers” is here. Supplements have become a necessary part of conducting regular scientific business, both from the standpoint of the original researcher in presenting their research in its entirety, and also to allow others to effectively use the original research.

The proposals herein represent only some of the changes necessary to maintain the usefulness of supplemental data. Outstanding concerns remain relating to the editing and peer review of these behemoths. As they become an integral part of science, detailed review of supplements will be increasingly necessary. One useful tactic may be detailed sampling: perhaps it is best for the editor to organize a system wherein, randomly, referees are asked to review samples in greater detail to ensure the overall quality of the supplements without quickly overwhelming the peer review system.

## Additional files

Additional file 1: Supplement to the main text. This file contains a document describing in more detail the discussion of the main text. (DOCX 62 kb)

Additional file 2: Figures S1. A and B from the supplementary text. (PDF 660 kb)

## Abbreviations

DOI: Digital object identifier
