# Supplementary material for: Structuring supplemental materials in support of reproducibility
Source: Genome Biol. 2017 Apr 5;18:64. doi: 10.1186/s13059-017-1205-3 (PMC5382465; doi:10.1186/s13059-017-1205-3)
Supplement: Supplementary file 1 — Supplement to the main text. This file contains a document describing in more detail the discussion of the main text. (DOCX 62 kb) [file 13059_2017_1205_MOESM1_ESM.docx]

**Supplement**

# Σ.I.VL Introduction (vernacular language)

There are a number of ways to share and preserve scientific data. These range in data-density from a simple citation in a research article, to a relatively lightweight abstract, to the more informative journal article, through to vast databases of scientific data. An emerging area within this spectrum, the Supplement, enables further discovery by tying the published results to their underlying data, and provides an important resource to enable reuse of the research by the broader community. However, in the aforementioned spectrum of scientific information density, supplements tend to fall far from the vernacular-friendly journal article, and more toward somewhat unreadable raw data.

Research papers are limited by journals’ physical and digital space constraints. Often, the more prestigious the journal, the more space restrictions are forced upon the authors. In a similar vein, the prestige of the journal can be used as leverage against an author to force them to otherwise present their data in a sub-optimal fashion, i.e., tersely, densely and without important details.

As a result of these and other limitations, and perhaps also in an effort to include research that has not yet been published elsewhere but needs to be presented for the authors to claim their conclusions before peers, many have turned to supplements as an alternative to mainstream publishing.

However, while scientific journals provide a useful presentation of data, having standardized headings that are used by all papers, regardless of the field, often unstructured supplements may not easily fit within the standards of the scientific publishing world, are not always peer reviewed, and are often not indexed [1]. Thus, although much scientific literature is highly structured and professionally indexed, supplements – perhaps one of the largest repositories of scientific information – lack both the structure and the indexability of the standard literature. At minimum, this results in data that is very hard to find, if not lost. At worst, it allows non-peer reviewed or even problematic data to leach into the scientific record through an unguarded back-door.

This is a problem that this paper sets out to solve.

# Σ.I.HL Introduction (high level)

As supporting materials become regular components of the record, questions are emerging regarding archiving useful versions of these materials. Part of the difficulty in doing this is that supplement data and information often take varied forms; data, software, algorithm descriptions, figures, or additional methods and text, to name a few. Each of these modalities requires documentation, curation, archiving, and preservation.

However, while “mesoscale” journal article supplements have become an increasingly indispensable resource for research – for presenting the full extent of their research, but also providing documentation and even repositories of scientific information and data – they are also failing to adequately do this job.

Given the essential nature of the supplement for scientific progress, and the general lack of coherent organization, recent efforts have sought to develop substantial journal oversight in this heretofore-unstructured area of publication. In this article, we propose a novel structuring of the Supplement section to bring coherence to this vital information source, and enable scholarly activities such as the verification of findings, reuse of data or software, and a more complete exposition of methods.

# Σ.I ∦A The supplement section today

This section, as denoted by the capital I∦A, does not directly parallel a subsection in the primary text (in the alternative, supplemental subsection Σ.I∥A *would* parallel subsection I.A in the main text). Nevertheless, this section does provide additional introductory material that might be of interest to the reader, and as such, is often included within the supplement. This section is also of use to the reader as it introduces terminology that might be usefully elucidated in a glossary section, including the definition and provision of integral content, and additional and associated content.

Even with their problems, supplements are valuable. In its current form, typically online publication of supplements provides an important and dedicated space for related and relevant information that simply will not fit within the limitations of a particular print or online publication [2, 3].

Moreover, given the massive scale of many current scientific efforts, the ability to reproduce and verify research results requires access to content not typically found in a journal article. This information could be contained in the supplemental materials, and could include all forms of supporting data and information relating to workflow and computational efforts. In particular, workflow and computational details frequently represent scientific decision making and assumptions that, if they were open to scrutiny, could improve the scientific process and allow follow-on researchers to better extend the results of the originally published research.

This supporting data and information can be divided into at least two types. Firstly, information that may be integral content [4]; for example, as well as allowing more text, supplements also provide space for oversized items such as tables, equations, figures, and high resolution images, or even unconventional items such as multimedia.

Secondly, the supplement allows the inclusion of additional or associated content; i.e., material that typically falls outside of standard presentation formats or their publication conventions, and which serves to provide context and further relevant explanations or background. These materials may include clarifying notes, data, software and its accompanying notation, workflows, failed experiments or negative results, and additional multimedia content [5, 6].

The use of supplements to provide access to all forms of the underlying raw data will become ever more relevant as supplements are used to fulfill journal requirements for the disclosure of that underlying data [7, 8].

Unfortunately, we believe that with much of this information residing in its current unstructured state, vast amounts of information stored in and represented by the publication may be undiscoverable, unusable, and unintentionally ignored. As such, supplements must be elevated to a standard publication form of research dissemination.

Supplements remain a form of publication, and like the corresponding primary paper, they need to be inherently representable in the standard publishable form: on printed paper. While some aspects proposed herein may seem less than optimally presented in a printed versus digital format, they all remain printable.

# Σ.I ∦B Glossary

This section provides a glossary for the terminology that is provided in the previous section.

“Integral content” relates to data that optimally should be included within the primary text if not for space limitations. “Additional” or “associated content” is material that typically falls outside of standard presentation formats, including, data provenance, glossaries such as this one, background information, workflows, and software-related information.

# Σ.I ∦C Current Concerns with Supplements

Notwithstanding the many positive aspects of supplements, many journals find their size and nature overwhelming. Some publishers are even calling for curbs in their use [9, 10]. Here, the supplement provides more citations than are provided the in primary paper [11–15], and it can also include atypical sources such as blogs and webpages.

Supplements often contain a tremendous amount of data, facts, and analysis, sometimes tenuously associated with their corresponding published papers. Standardization will help prevent this additional information from getting lost. In some instances, references to the Wild West have been made in characterizing the current status quo for supplemental material [16]; for example, with some otherwise short papers, supplemental materials can be nearly 30 times their length [11, 17]. We believe that these and other issues can be addressed with a more considered approach to supplemental materials, to be described herein.

Efforts to rein in supplements are necessary on a more practical level as well. As a result of the often disorganized nature of current supplements, authors regularly cram as much information as possible into the main text of the document, making it unreadable by overloading the limited space with too much data in lieu of tersely written vernacular text. Although much of the rest of the data will be dumped into the supplement, supplements often lack extensive editing and mincing, making finding the relevant data, in both the primary text and the supplement ever more difficult.

Even with these concerns, many journals support and even promote the extensive use of supplements [8]. Broad efforts, such as this one, continue to be made to establish a set of best practices to address several aspects related to supplemental materials [18].

# Σ.II Proposal: standardizing the supplemental materials section for genomic research articles

Examples of best practice for supplements ought to be designed to deal with the above-mentioned concerns, as well as other issues pertinent to supplemental material. These examples should include guidelines relating to size and format (including documentation), scope, persistence, and accessibility of supplemental material.

Additional examples of best practices should relate to the curatorial responsibility of journals, focusing on remedying the general lack of peer review and discoverability, and the inability to cite substantial portions of the information found only in the supplementary materials.

Ours is not the first effort to suggest better administration of supplements [2, 9, 19-20]. However, several concerns specific to genomic-oriented journals have been otherwise overlooked in other efforts, particularly in the areas of interoperability, interpretability, reusability, organization, versioning, granularity in large dynamic data sets, and overall standards. With the growing relevance and importance of supplemental materials in genomic research, we propose several additional changes that can be employed in the publication of supplements to help make the information published therein more useful for the researcher.

With a recognized and useful supplement, such as this one, authors need not attempt to fit as much raw data and tenuously related information into the paper, and as such, the main text can be made more readable. This is particularly the case if each section and subsection in the main text can be directly tied to the corresponding expanded section or subsection through an established, logical, and linked hierarchy.

Even though the supplement will likely never be as refined a document as the main text, improvements can be made.

# Σ.II.TL Proposal (technical language)

1. Digital object identifiers (DOIs), micro-referencing, and hierarchical headings
2. Supplements should fit within the research stack of an archived of scientific record
3. Supplements should include workflows, data verification and provenance, and should be curated
4. Supplements should be designed to be findable, crawlable and readable by indexes such as Web of Science, Pubmed and Google Scholar
5. Supplements should follow FAIR (findable, accessible, interoperable and reusable) standards

# Σ.II.CPL Proposal (computer-parsable language)

1. Attribution: DOI; micro-referencing
2. Design: hierarchical headings
3. Elements: workflows; data verification and provenance; subsections
4. Search: findable; crawlable; readable; indexed

Qualities: FAIR; readable; structured; printable

## Σ.II∥A proposed hierarchy

In this proposed hierarchy, the primary text sits atop the supplement, synthesizing the supplemental information in broad strokes. Local links point to more detailed descriptions of methods and data located further within the supplemental materials. For example, the supplement would include a detailed description expanding upon the top-level primary text, which would be logically divided so that each division addresses one coherent aspect of the analyses. The order of these divisions would map onto the order of appearance within the top-level primary text. Additionally, the divisions would also map onto the published paper, allowing researchers to easily move between the supplement and the original paper. As a bonus, a clearer hierarchy that can be easily mapped onto the original published paper will make adding, editing, or modifying these links, whether internally and externally, that much easier.

In a secondary hierarchical structure, each of these individual divisions may relate to its own, huge number of supplementary calculations and data sets. These calculations and datasets would be further linked such to relate back to each division within the top-level primary text. Moreover, to promote machine readability of the data sets, data could optimally be provided in a standard tabular format, for example comma-separated values (CSV). Charts, graphs and other pictorial representations of the data should be decomposable, for example accompanied by machine readable files comprising the underlying images.

Practically speaking, all data falling within the hierarchy should be localized to a single digital location. When necessary, hyperlinks can be provided to outside sources, but all supplemental data should fall within the scope of the supplement section of the journal.

In some cases, the sheer size of intermediate or non-essential data sets may require some data to reside in an off-site website, provided that the authors guarantee viability to the links. Here, usage of standard, widely accepted repositories, for example, an institutionally supported and persistent website, a commercial cloud, or even a shared community repository, might be best. (See **Σ.Figure**∥1)

## Σ.II∥B Hierarchical information structures

To understand the overall structure of the supplement, one has to think of scientific writing both in terms of a hierarchy and parallel passes at increasingly greater levels of detail. Supplements can be both. They can provide a hierarchy in the sense that they divide the information into discrete chunks so that readers can avoid reading through a tremendous amount of highly detailed yet potentially irrelevant (to their present interests) text. Additionally, a hierarchy provides a roadmap: reading a scientific text can be seen as analogous to an information retrieval task, wherein a reader first peruses an introductory idea section and then jumps into a more detailed version of that section. The current structure of a standard scientific manuscript implements a long-standing version of this idea. A vague yet still informative title, a more detailed abstract, a somewhat expanding introduction, a detailed results section with even more detailed tables, and then – moving back out – a conclusion that applies the details therein more broadly. The proposed supplement guidelines would expand on this age-old structure, building onto this pre-existing hierarchy and providing even more detail.

This hierarchical structure would operate in a parallel fashion to the main text. Essentially it would be a shadow text that directly tracks and corresponds to the main text, providing more detailed explanations for each heading. A reader looking for more detail on a particular part of the main text could easily find and then consult the analogous part of the supplement, which would be similarly situated within the hierarchical structure. Employing an apt literary metaphor: the published paper represents primary classical textual sources, and the supplement mirrors the annotation, gloss and other editorial or academic content on that original text, adding integral, associated, and tangentially relevant context. However, the versatility of the supplement allows it to be more than simply analogous to the annotator’s close elucidation on a Shakespearian sonnet; it can also be useful at the other end of the spectrum as an expansive and sometimes meandering – albeit hierarchically organized – Talmud to the Torah of the succinctly and sometimes cryptically presented published paper. Notably breaking with these metaphors however, supplement authors are also the original authors who may present otherwise unseen connections and information. Further extending the metaphor, the author of the supplement can act as a curator and editor of a collection of works, providing relevant information to present unseen connections across the body of work, for example. See, e.g. Σ. ∥Fig.1.

This hierarchical mirroring can be readily extended to the figures and tables, which can be more detailed in the supplement. The idea of both a hierarchy with increasing levels of detail, and a parallel text shadowing it, can be extended beyond a single paper to a whole collection of papers – as is often the case in a large multigroup project where a coauthored high level paper describes the overall structure of the project, and a succession of more detailed papers (often across multiple journals) describes single, specific, drilled-down ideas. With ‘big consortia science’ projects publishing multiple interconnected papers, a global hierarchy for all related papers can be developed, with that global hierarchy corresponding to various supplements associated with papers published in conjunction with a primary roll-out, or even subsequent papers. This system would also give a clearer picture of the interconnectivity of individual papers.

This proposed hierarchy would include standardized headings for easy human and machine readability, with the structured headings directly corresponding to headings in the primary paper. Additionally the supplementary material should be designed to include ample, indexable metadata linking various elements within the hierarchy of the paper.

## Σ.II ∥C The FAIR standards: findable, accessible, interoperable, and reusable

As provided in the primary paper, the recent FAIR approach for scientific information relates to both human and machine analysis of presented data [21]. For example, with regard to the FAIR standards, see also [22–26]. For similar ideas, see [27, 28]. (Noteworthy of this supplement, we provide additional citations to the primary text.)

Succinctly, under this paradigm, scientific data in supplementary material should be: findable, accessible, interoperable and reusable.

Data should be findable both for human researchers as well as computers, requiring unique and persistent identifiers; for example those provided by groups such as Consortia Advancing Standards in Research Administration Information (CASRAI) [29] for the data and its associated components (e.g., metadata and documentation).

Data ought also to be accessible. Here, accessibility relates mainly to good data stewardship, and in particular, data, code, and workflow information should be stored long-term, and be legally accessible via appropriate open licensing and other methods necessary for non-inhibiting access [30, 31].

Accessibility also relates to making the underlying software code accessible. However, while supplemental material should always strive to provide all relevant information in one place, including a snapshot of the version of the software code used for the analysis, subsequent and further evolving versions of the code should be linked to – perhaps even indexed – but stored separately. Specialty sites such as GitHub or BitBucket may be used for this, provided that adequate metadata is included so as not to inhibit the discovery of relevant software [32]. To this end, software versioning is vital for reusable and changeable objects like data and software, and DOIs should be assigned to all data, code, and workflow information associated with the published findings [33].

Data stored in supplements should also be interoperable, as human readers need to clearly understand the connection between the data and the main text. Readers should be able to appreciate the nature of the data from its presentation, particularly how it can be combined or compared with other data sets. Interoperability requires that the data also be easily digested by computational systems, using a standard that allows straightforward data manipulation.

Finally, data needs to be reusable. Both humans and machines should be able to apply the data to follow-up research and additional computational analysis.

**Σ.II** ∥D **Provenance**

With the growth of “Big Data”, there is an increasing need for veracity and verifiability of research data. The provenance of data refers to a complete description of its origins, as well as how it arrived in its current database and current form (by conversions, normalizations, etc.) Succinctly: data should be tracked as it is collected and repackaged in subsequent research [34].

Provenance is highly relevant to assessing data quality, which can often be estimated based on the source of that information. Effectively, this (i) provides an audit trail that will allow appreciation of the resource usage in putting together the dataset; (ii) locate the potential source of any errors in the data; (iii) provide the location of all the data relevant to replicate the results; and, (iv) provide attribution of the resulting data and conclusions. This is an important issue for assessing ownership, copyright, license limitations, any privacy restrictions, and liability, if any, ascribed to erroneous data.

## Σ.II∥E Workflows

Supplementary material should be designed to incorporate workflow-related information. For example, the supplement can outline the individual and collective workflows that resulted in the eventual data set, and the published conclusions [35]. Workflows are especially relevant for *in silico* analyses, as the exact particulars and parameters employed can make all the difference between reproducible and non-reproducible data. In this regard, supplemental data should include both abstract versions of workflows, as well as flowcharts or similar representations of the executed workflows as they relate to the particular code and execution infrastructure of the lab conducting the research [36].

Workflows should be directly linked to specific figures and files associated with the paper so that subsequent researchers can review and analyze the transformations, analyses, or other manipulations that have already been applied to a data set. Similarly, subsequent researchers can understand the implemented processes that resulted in the figure, from raw data to processed data, to a supplemental table of the processed table, to a figure in the primary paper, and finally to the text describing that figure.

Workflows should also have their own standardized identifiers, such that those identifiers include references to the relevant datasets associated with the workflow, any relevant software applied to the workflow, dates that further help to describe the version of the data and the software, and any other relevant information that could be used to cross-reference different data sets and their associated workflows. In the alternative, third party solutions such as Galaxy [37] could be used to organize workflows. The supplement can include links to such solutions [38].

## Σ.II∥F Language in the supplement

A key aspect of scientific writing is language. The nature of scientific progress and the evolution of myriad micro-disciplines have resulted in scientific writing that can be difficult to understand. To some degree, this jargon-filled language can be justified, as it offers the necessary precision to properly present research, reproduce a result, and to effectively automatically parse through text. On the other hand, the broader scientific community would likely appreciate a simpler, more vernacular language that is easier for a generalized audience to understand; one that is potentially more communicative, allows cross-disciplinary fertilization, and perhaps better reflects the multidisciplinary nature of many current scientific efforts.

The “Goldilocks problem” of finding the level of jargon that is necessary for accuracy but does not alienate the broader readership could potentially be overcome through the effective use of supplements. Overall, the supplement allows multiple “languages” that are easily understood by human researchers, as well as being machine-readable. In some instances, this might be reflected in a standardized hierarchy and standard terminology; in other cases it may necessitate otherwise awkwardly composed machine-readable text juxtaposed to human-readable text. The supplement can contain a jargon-free schematic of the research, or easy-to-understand presentations or graphics that an author might use in a scientific or lay presentation. While the basic information in these graphics is likely not suitable for the main text, it remains extremely valuable in terms of communicating ideas to broader audiences.

This merger of presentation material with publication material has obvious benefits: for example, a standard conference talk presenting a paper typically contains important background information, and even historical or scientific context that is often not included within the introductory sections of the published paper. Including this information within a supplement is likely to be of substantial value to researchers from other fields. Further, providing additional components of the slide deck from a talk, or even several related presentations, could effectively merge a dynamic presentation of the data with its heretofore more static published presentation. As with data and other digital artifacts, a DOI should be assigned to an associated slide deck.

We believe it is essential that vocabularies, taxonomies, and metadata be standardized such that data can be easily read and manipulated across labs, fields, and time. To this end, the supplement could also have a very precise glossary – described briefly above – translating language used in the paper into precise database identifiers and standardized names so that machine text miners can learn how to easily parse through a supplement and relate it to a database entry.

**Σ.II**∥**G** **Citation standards**

With an established hierarchy, different components of the paper and its supplement can be referenced intelligently, including distinct DOIs for portions of the paper itself, as well as related identifiers, through the clever use of prefixes and suffixes for related portions within the supplement. The use of DOIs need not be limited to text, but can be expanded to include suffixes for related figures, tables, data sets, and other related information. DOIs, or similar systems, would also be useful given the nature of the supplement, allowing information to be inserted or deleted without otherwise complicating the retrieval of other information. This use of DOIs is especially important in large supplements for which it would be too time consuming to find the desired section, text, figure, or other source of information. Here, simply directing the reader to the supplement – as is, unfortunately, all too common – would effectively be a fool’s errand without micro-referencing.

Unlike the published text, authors can take advantage of the nature of the supplementary section to micro-reference micro-authorship, utilize open researcher and contributor IDs (ORCIDs), or other persistent unique identifiers to uniquely identify authors and note who contributed to each portion of the paper, whether those authors appeared on the original publication or not. Not only would this more realistically accredit authors than standard author listings, but it would give interested readers direct access to each author responsible for the particular area, text, figure of interest, perhaps through published email addresses.

Figures would not only include captions and links to relevant parts of the text, but might also include additional information related to the relevant contact individuals for each figure, and access to the source code and data that generated the figure. Again, this would be particularly important given the growing trend to list tens if not hundreds of authors on biological papers.

Supplementary material will also include an expanded bibliography, which can be designed to provide contextual information, both with regard to the paper itself and the supplementary material. Furthermore, the bibliography can be annotated to provide substantive information as to how each source relates to the presented information.

Citation standards should be broadened to allow pinpointed referencing between the primary text and the supplemental text, such that readers of the primary text will be directed from the main text to the relevant section in the supplemental material, and readers of the supplemental material will be directed back to the relevant portion of the main text. Micro-DOIs or other reference systems might be used for this. To some degree, micro-referencing can be accomplished through an elegant hierarchical structure in the main text, which would be shadowed in the supplemental text and/or vice versa. This should be further simplified through a standardized numbering system, allowing sections, subsections, and even further divisions if necessary.

Further, this citation standard can include additional information relating to super-sections, tying together published papers across multiple journals and even disciplines. Optimally, publication databases would provide identifiers to not only the main published paper, but would at minimum list the other identifiers associated with the paper.

# Σ.III ∥Conclusions

Supplements have become a necessary part of regular scientific business, both from the standpoint of the original researcher in presenting their research in its entirety, and also from that of the follow-up researcher to effectively use the original research.

Although we provide a comprehensive wish list for a supplement to deal with the many issues inherent in current supplementary materials, concerns remain relating to the editing and peer review of these behemoths. As they become an integral part of science, detailed review of supplements will be increasingly necessary; however, given their large size, one approach may be to review random samples of the supplement, or to utilize a trusted third party such as ResearchCompendia.org [39] to verify computational results.

The popularity of consortia science and the deluge of data it brings have created an ever-growing need for more structured supplemental data. This is necessary not only for providing FAIR access to important data sets, but also for the increasing use of machine learning tools to mine scientific literature. The proposals herein represent only some of the changes necessary to maintain the usefulness of supplemental data.

**References**

1. Hopewell S, Clarke M, Mallett. Grey literature and systematic reviews. In: Rothstein HR, Sutton AJ, Borenstein M, editors. Publication bias in meta-analysis: prevention, assessment and adjustments. Chichester, UK: Wiley; 2005. p. 48–72.

2. Borowski C. Enough is enough. J Exp Med. 2011;208(7):1337.

3. Pachter L. Stories from the supplement. 2 November 2013 [cited 12th December 2016]. In: Bits of DNA [Internet]. Available from: https://liorpachter.wordpress.com/2013/11/02/stories-from-the-supplement/.

4. National Information Standards Organization. Recommended practices for online supplemental journal article materials [Internet; accessed 12th December 2016]. Baltimore, MD, USA: National Information Standards Organization; 2013. Available from: http://www.niso.org/apps/group_public/download.php/10055/RP-15-2013_Supplemental_Materials.pdf.

5. Kenyon J, Sprague NR. Trends in the use of supplementary materials in environmental science journals. Issues in Science and Technology Librarianship. 2014. doi: 10.5062/F40Z717Z.

6. Stodden V. Resolving irreproducibility in empirical and computational research. IMS Bulletin [Internet]. November 2013 [cited 12th December 2016]. Available from: http://bulletin.imstat.org/2013/11/resolving-irreproducibility-in-empirical-and-computational-research/.

7. Taichman DB, Backus J, Baethge C, Bauchner H, de Leeuw PW, Drazen JM, et al. Sharing clinical trial data: a proposal from the International Committee of Medical Journal Editors. Lancet. 2016;144(1):11–3.

8. Hanson B, Sugden A, Alberts B. Making data maximally available. Science. 2011;331(6018):649.

9. Maunsell J. Announcement regarding supplemental material. J Neurosci. 2010;30(32):10599–600.

10. Marcus E. Taming supplemental material. Cell. 2009;139(1):11.

11. Pop Mihai, Salzberg SL. Use and mis-use of supplementary material in science publications. BMC Bioinformatics*.* 2015;16:237.

12. Seeber F. Citations in supplementary information are invisible. Nature. 2008;451(7181):887.

13. Weiss MS, Einspahr H, Baker EN, Dauter Z, Kaysser-Pyzalla AR, Kostorz G, et al. Citations in supplementary material. Acta Crystallogr Sect F Struct Biol Cryst Commun. 2010;66(Pt 12):1550–1.

14. Rafferty AR, Wong BB, Chapple DG. An increasing citation black hole in ecology and evolution. Ecol Evol. 2015;5(1):196–9.

15. Kenyon J, Sprague N, Flathers E. The journal article as a means to share data: a content analysis of supplementary materials from two disciplines. Journal of Librarianship and Scholarly Communication. 2016;4:eP2112. doi: 10.7710/2162-3309.2112.

16. Carpenter T. Standards column – taming the world of data: pressures to improve data management in scholarly communications. Against the Grain [Internet]. 19 January 2011 [cited 12th December 2016]. Available from: http://www.against-the-grain.com/2011/01/v22-6-standards-column/.

17. Newton-Cheh C, Johnson T, Gateva V, Tobin MD, Bochud M, Coin L, et al. Genome-wide association study identifies eight loci associated with blood pressure. Nat Genet. 2009;41(6):666–76.

18. Schwarzman AB. NISO/NFAIS Supplemental Journal Article Materials Working Group: a progress report. Journal Article Tag Suite Conference (JATS-Con) Proceedings 2010. Bethesda, MD, USA: National Center for Biotechnology Information (US); 2010.

19. Carpenter T. Outside the core: working towards an industry recommended practice for supplemental journal materials. Serials. 2010;23(2):155–8. doi: [10.1629/23155](http://doi.org/10.1629/23155).

20. Moderating supplementary data. Nat Neurosci. 2012;15(3):339.

21. Force11.org [Internet]. Guiding principles for findable, accessible, interoperable and re-usable data publishing: version B1.0 [cited 12th December 2016]. Available from: https://www.force11.org/node/6062.

22. Wilkinson MD, Dumontier M, Aalbersberg IJ, Appleton G, Axton M, Baak A, et al. The FAIR Guiding Principles for scientific data management and stewardship. Sci Data. 2016;3:160018.

23. Rodríguez-Iglesias A, Rodríguez-González A, Irvine AG, Sesma A, Urban M, Hammond-Kosack KE, et al. Publishing FAIR data: an exemplar methodology utilizing PHI-Base. Front Plant Sci. 2016;7:641.

24. Da Silva Santos, Luiz Olavo Bonino, et al. FAIR Data Points Supporting Big Data Interoperability. In: Zelm M, Doumeingts G, Mendonça JP. Enterprise Interoperability in the Digitized and Networked Factory of the Future, ISTE Press; 2016. p. 270-279.

25. Wilkinson MD, Verborgh R, Bonino da Silva Santos LO, Clark T, Swertz MA, Kelpin FDL, et al. Interoperability and FAIRness through a novel combination of web technologies. PeerJ Preprints. 2016;5:e2522v2.

26. Rodríguez Iglesias, Alejandro, et al. "Publishing FAIR Data: an exemplar methodology utilizing PHI-base." Frontiers in Plant Science 7 (2016): 641.

27. McQuilton P, et al. BioSharing: curated and crowd-sourced metadata standards, databases and data policies in the life sciences. Database (Oxford). 2016;2016: baw075.

28. Nichols TE, Das S, Eickhoff SB, Evans AC, Glatard T, Hanke M, et al. Best practices in data analysis and sharing in neuroimaging using MRI. Nat Neurosci. 2017;20(3):299–303.

29. Haak L, Baker D, Höllrigl T. CASRAI and ORCID: Putting the pieces together to collaboratively support the research community. Procedia Computer Science. 2014;33:284–8.

30. Stodden V. Enabling reproducible research: licensing for scientific innovation. International Journal of Communications Law and Policy. 2009;13:1.

31. Donoho DL, Maleki A, Shahram M, Ur Rahman I, Stodden V. Reproducible research in computational harmonic analysis. Computing in Science and Engineering. 2009;11(1):8–18.

32. Da Veiga Leprevost F, Barbosa VC, Francisco EL, Perez-Riverol Y, Carvalho PC. On best practices in the development of bioinformatics software. Front Genet. 2014;5:199.

33. Stodden V, Miguez S. Best practices for computational science: software infrastructure and environments for reproducible and extensible research. Journal of Open Research Software. 2014;2(1):e21. doi: 10.5334/jors.ay.

34. Bechhofer S, Buchan I, De Roure D, Missier P, Ainsworth J, Bhagat J, et al. Why linked data is not enough for scientists. Future Generation Computer Systems. 2013;29(2):599–611.

35. Donoho D, Stodden V. Reproducible research in the mathematical sciences. In: Higham NJ, editor. Princeton companion to applied mathematics. Princeton, New Jersey: Princeton University Press; 2015. p. 916–24.

36. Garijo D, Gil Y. A new approach for publishing workflows: abstractions, standards, and linked data. Proceedings of the 6th Workshop on Workflows in Support of Large-scale Science; 2011 Nov 12–18; Seattle, WA, USA. ACM; 2011.

37. GalaxyProject.org [Internet; accessed 12th December 2016]. Available from: https://galaxyproject.org/.

38. Deelman E, Gannon D, Shields M, Taylor I. Workflows and e-Science: an overview of workflow system features and capabilities. Future Generation Computer Systems. 2009;25(5):528–40.

39. ResearchCompendia.org [Internet; accessed 12th December 2016]. Available from: http://www.ResearchCompendia.org.
