# Supplementary material for: Structuring supplemental materials in support of reproducibility
Source: Genome Biol. 2017 Apr 5;18:64. doi: 10.1186/s13059-017-1205-3 (PMC5382465; doi:10.1186/s13059-017-1205-3)
Supplement: Supplementary file 2 — A and B from the supplementary text. (PDF 660 kb) [file 13059_2017_1205_MOESM2_ESM.pdf]

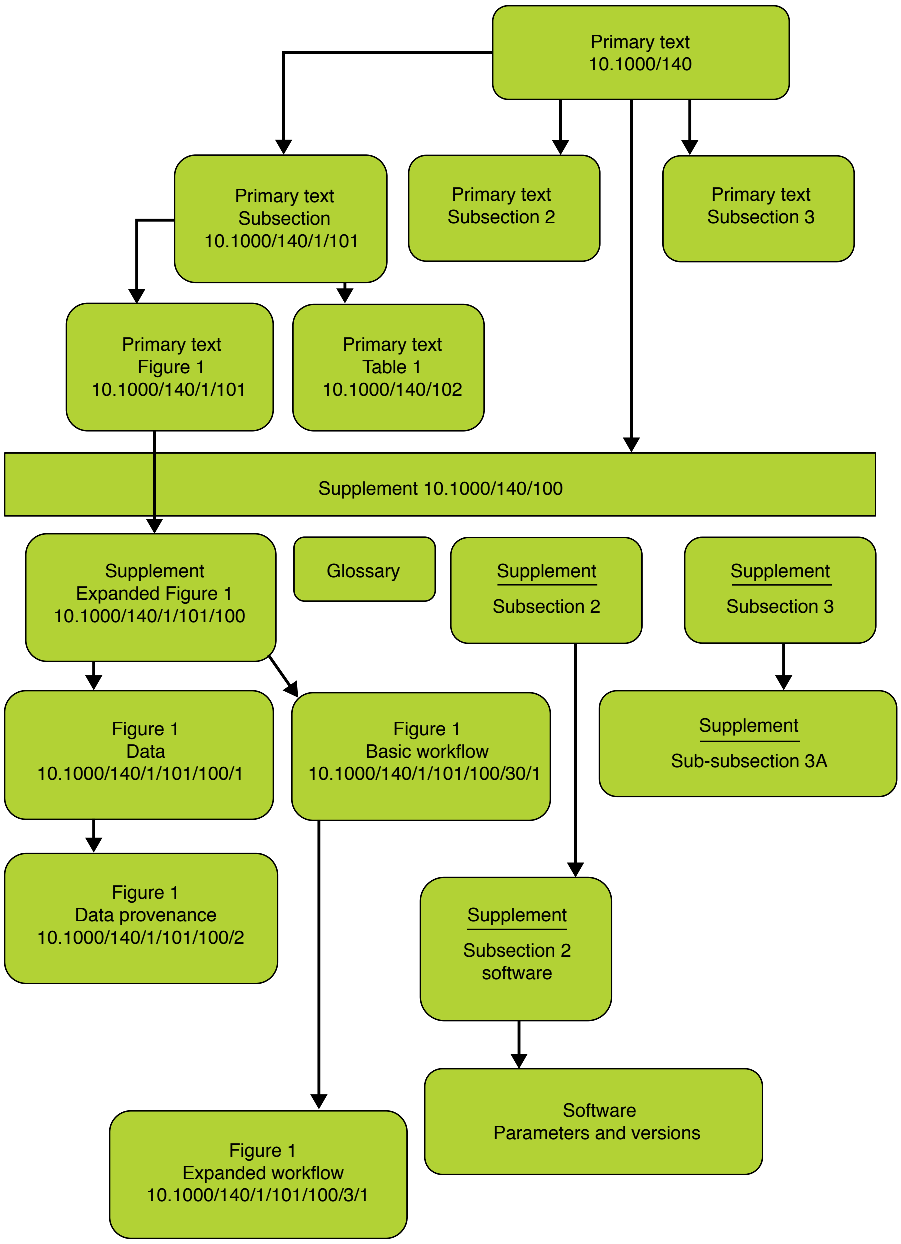
Primary text
10.1000/140
Primary text
Subsection
10.1000/140/1/101
Primary text
Subsection 2
Primary text
Subsection 3
Primary text
Figure 1
10.1000/140/1/101
Primary text
Table 1
10.1000/140/102
Supplement 10.1000/140/100
Supplement
Expanded Figure 1
10.1000/140/1/101/100
Glossary
Supplement
Subsection 2
Supplement
Subsection 3
Figure 1
Data
10.1000/140/1/101/100/1
Figure 1
Basic workflow
10.1000/140/1/101/100/30/1
Supplement
Sub-subsection 3A
Figure 1
Data provenance
10.1000/140/1/101/100/2
Supplement
Subsection 2
software
Software
Parameters and versions
Figure 1
Expanded workflow
10.1000/140/1/101/100/3/1

Σ. Fig. ∦1A

Σ.Fig.1A is a supplement to Fig. 1 in the primary text. Like the supplement itself, Σ.Fig.1 expands on the information provided in Fig.1, in fashion paralleling Fig. 1. For example, for each subsection, the figure shows a digital object identifier (DOI) that identifies each section, and like the primary text itself, is indexed. In addition to the DOI, each section should also provide one or more authors and their contact information. Supplements may also include, in addition to the elements provided in Fig. 1, a glossary, as well as non-parallel sections, as shown in Σ.Fig.1. Further, the supplement figure indicates that supplements will provide additional relevant background information for tables and figures provided in the primary text, including data provenance, software, schematic workflows, and more expanded workflows. Software used in the primary text may be better described in the supplemental text, including descriptions of the parameters employed, and a software version number. The supplement may also contain descriptions of failed research efforts, such that negative results are also provided, not simply the positive results that are often given in the primary text.

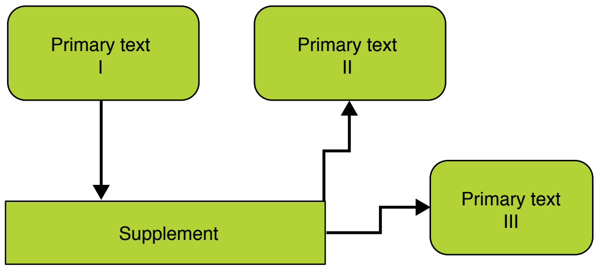
Primary text I
Primary text II
Primary text III
Supplement

Σ. Fig. ∦1B

Primary Texts I, II and III may be related papers; for example, part of a rollout from a consortium study. Alternatively, Primary Texts I, II and III may be substantially unrelated but have some related components; for example, they may share a data set or workflow. Primary Texts I, II and III may represent successive efforts by the same or different groups in a research endeavor. Primary Texts I, II and III may be otherwise seemingly unrelated. Here, the Supplement can present connections that are obvious or not, parallels and relationships between the papers, thus providing greater context for the audience.
